# IL‐38: A novel cytokine in systemic lupus erythematosus pathogenesis

**DOI:** 10.1111/jcmm.15737

**Published:** 2020-10-20

**Authors:** Wang‐Dong Xu, Lin‐Chong Su, Xiao‐Yan Liu, Jia‐Min Wang, Zhi‐Chao Yuan, Zhen Qin, Xi‐Ping Zhou, An‐Fang Huang

**Affiliations:** ^1^ Department of Evidence‐Based Medicine Southwest Medical University Luzhou China; ^2^ Department of Rheumatology and Immunology Minda Hospital of Hubei Minzu University Enshi China; ^3^ Department of Rheumatology and Immunology Affiliated Hospital of Southwest Medical University Luzhou China

**Keywords:** autoimmunity, IL‐38, inflammation, lupus

## Abstract

IL‐38 is a newly identified cytokine that belongs to the IL‐1 family. In our previous study, we found elevated plasma levels of IL‐38 in patients with systemic lupus erythematosus (SLE). However, the clear relationship of IL‐38 expression in plasma, peripheral blood mononuclear cells (PBMCs) and clinical and laboratory features needs elucidation. Additionally, we evaluated the possible role of IL‐38 in regulating production of inflammatory cytokines in PBMCs in vitro. A pristane‐induced murine lupus model was used to further demonstrate the effects of IL‐38 on cytokines in vivo and discuss the significance of IL‐38 in lupus development. The results showed that mRNA expression of IL‐38 in PBMCs of patients with SLE was elevated compared with volunteers, and expression of IL‐38 in both plasma and PBMCs was strongly related to clinical features, such as haematuria and proteinuria, and correlated with a SLEDAI score. Plasma levels of TNF‐α, IL‐1β, IL‐6 and IL‐23 were elevated in patients with SLE and were related to plasma levels of IL‐38. In vitro, PBMCs of patients with SLE stimulated with IL‐38 showed a decreased expression of the four inflammatory cytokines compared with PBMCs of patients without treatment. Interestingly, IL‐38 administration in lupus mice significantly reduced the development of lupus, such as reduced proteinuria, improved histological examinations of the kidneys and down‐regulated inflammatory cytokines. In conclusion, IL‐38 may suppress synthesis of pro‐inflammatory cytokines and therefore regulate lupus pathogenesis.

## 1 INTRODUCTION

Systemic lupus erythematosus (SLE) is a complex, inflammatory autoimmune disorder. Genetics and environmental factors interacted in occurrence of the disease. It is characterized by dysregulation of autoantibodies, complement system and inflammatory cytokines.^1^ Cytokine‐mediated immunity has been found to perform significantly in SLE pathogenesis.^2^, ^3^

Interleukin‐38 (IL‐38, previously called IL‐1F10) belongs to the IL‐1 family and has been identified in recent years. The human IL‐38 gene has 4 exons and is located within chromosome 2q13‐14.1 near IL‐1Ra and IL‐36Ra.^4^ IL‐38 has a weight of 17‐18 kDa. It is expressed in several immune cells such as B cells, monocytes and macrophages and some tissues, including the skin, spleen and thymus.^5^, ^6^ IL‐38 binds to receptor chains, IL‐1R1, IL‐36R and IL‐1RAPL1,^7^, ^8^ and then induces an immune response. Recent findings have shown that skin and serum levels of IL‐38 were reduced in psoriatic patients,^9^ whereas IL‐38 expression in patients with Sjogren's syndrome (SS) was higher as compared with controls.^10^ Interestingly, IL‐38 concentration was elevated in serum from patients with SLE.^11^ In our previous study, patients with SLE revealed higher plasma levels of IL‐38.^12^ To further discuss the relationship of IL‐38 and lupus, we first elucidated the association of IL‐38 expression in plasma, peripheral blood mononuclear cells (PBMCs) with disease activity, and clinical and laboratory characteristics. We then discussed the role of IL‐38 in the production of inflammatory cytokines both in vitro and in vivo, as well as the significance of IL‐38 in lupus development.

## 2 METHODS

### 2.1 Patients with SLE

Forty‐one newly diagnosed patients with SLE (37 females and four males, median age: 43.0 [26.5‐54.5] years) fulfilling the American College of Rheumatology revised criteria for SLE were recruited.^13^ Thirty‐one age‐ and sex‐matched volunteers were assigned to be normal controls (27 females and four males, median age: 40.0 [37.0‐49.0] years). Disease activity was assessed for each individual patient with the SLE Disease Activity Index (SLEDAI).^14^ All the participants gave informed consent according to the Declaration of Helsinki. The current research was approved by the Ethics Committee of Southwest Medical University. Serological evaluation that included a test for antibodies and complements and erythrocyte sedimentation rate (ESR) was performed. Clinical and laboratory data of individual patient were recorded, and the information is summarized in Table 1.

**TABLE 1:** Demographic, clinical and laboratory characteristics of SLE patients and healthy controls

Characteristics	SLE (N = 41)	Healthy controls (N = 31)	*P* value
Age (y)	43.0 (26.5‐54.5)	40.0 (37.0‐49.0)	>.05
Sex (male/female)	4/37	4/27	>.05
BMI (kg/m^2^)	22.3 (19.3‐25.1)		
Disease duration (y)	2.0 (0.0‐6.5)		
Arthritis (n, %)	14 (34.1)		
Rash (n, %)	8 (19.5)		
Alopecia (n, %)	12 (29.3)		
Pericarditis (n, %)	6 (14.6)		
Fever (n, %)	6 (14.6)		
Reduced platelet (n, %)	9 (22.0)		
Haematuria (n, %)	9 (22.0)		
Proteinuria (n, %)	13 (31.7)		
Pyuria (n, %)	11 (26.8)		
ANA (+) (n, %)	38 (92.7)		
Anti‐dsDNA (+) (n, %)	8 (19.5)		
Anti‐Sm (+) (n, %)	19 (46.3)		
Anti‐SSA (+) (n, %)	25 (61.0)		
Anti‐SSB (+) (n, %)	9 (22.0)		
Anti‐rRNP (+) (n, %)	13 (31.7)		
C3 (g/L)	0.73 ± 0.32		
C4 (g/L)	0.18 (0.05‐0.22)		
IgA (g/L)	3.56 ± 1.22		
IgM (g/L)	0.84 (0.54‐1.16)		
IgG (g/L)	14.70 (0.79‐19.54)		
ESR (mm/h)	48.75 ± 3.11		
SLEDAI	7.0 (3.5‐16.0)		

Abbreviations: BMI, body mass index; SLE, systemic lupus erythematosus; SLEDAI, SLE Disease Activity Index.

### 2.2 PBMC isolation and plasma preparation from patients

Venous blood (10 mL) from each participant was obtained and handled within 3 hours. The PBMC fraction was obtained by density‐gradient centrifugation. Isolated human PBMCs were washed twice in phosphate buffer saline and then used for cell culture or total RNA extraction. Plasma for each participant was stored at −80°C until determination.

### 2.3 Cell culture

PBMCs were fostered in RPMI 1640 supplemented with 10% FCS, 100 IU/mL penicillin and 100 μg/mL streptomycin (all from HyClone, Thermo) under the condition of 37°C and 5% CO_2_. The cells (5 × 10^5^ cells/mL/well) were first treated for the presence or absence of recombinant human IL‐38 at 200 ng/mL for 24 hours and then stimulated with lipopolysaccharides (LPS, 1 μg/mL) for 6 hours. Finally, supernatants were gathered and stored at −80°C until enzyme‐linked immunosorbent assay (ELISA) analysis.

### 2.4 Animal

Eight‐week female C57BL/6 mice were purchased from SPF Biotechnology. The mice were treated according to federal and institutional guidelines on animal welfare according to Animal Ethics Committee of Southwest Medical University and had free access to food and water in a temperature‐controlled room with a 12‐hour light/dark cycle. The mice were divided into three groups. Five mice received 500 uL saline intraperitoneally injected once. Ten mice received 500 uL pristane (Sigma‐Aldrich) intraperitoneally once, which were further divided into two groups at the 20th week after injection of pristane (5 mice/group): pristane‐induced lupus mice and pristane‐induced lupus + IL‐38 mice. The lupus + IL‐38 group was injected with recombinant murine IL‐38 (AdipoGen) for 7 days intravenously (iv) every day at the base of the tail (2.5 ng/uL), and the lupus group received saline (iv) daily for 7 days. All the mice were monitored and killed at the 24th week.

### 2.5 Biochemical and physiological characteristics of the mice model

Urinary protein and skin lesions were assessed monthly beginning at the age of 8 weeks. Urinary protein expression was determined freshly by collecting morning urine using a semi‐quantitative test for each mouse, evaluated as 0‐4 according to the manufacturer (Bayer Clinitek).

### 2.6 Histochemical staining

Two kidneys from each individual mouse were separately used for morphometrical and immunofluorescence assays and ultrastructural analysis.^15^ One kidney was fixed in 10% formaldehyde solution, dehydrated in ethanol and processed for paraffin embedding. Serial 5‐um tissue sections of each sample were cut. Haematoxylin and eosin (H&E) and Masson stain were used to check the pathological stage of the specimen tissue. The kidney tissue was stained with FITC‐conjugated anti‐mouse IgG (Abcam) for immunofluorescence analyses, and a fluorescence microscope (FV1000, Olympus) was used for observing distribution of collagen and immunoglobulin deposition.^16^ Another kidney was fixed in 2.5% glutaraldehyde and post‐fixed in 1% osmium tetroxide, dehydrated in acetone and embedded in Epon resin. The samples were cut with an ultramicrotome (EM UC7, Leica) obtaining 50‐nm‐thick sections and reviewed by transmission electron microscopy (JEM‐1400Plus, JEOL) after staining with uranyl acetate and lead citrate solutions.

### 2.7 ELISA for IL‐38, inflammatory cytokines, ANA and anti‐dsDNA

Plasma IL‐38 expression was tested by ELISA as previously described.^12^ In this study, we discuss the role of IL‐38 in PBMCs and mainly evaluated effects of IL‐38 on cytokines TNF‐α, IL‐1β, IL‐6 and IL‐23. Therefore, we assessed the plasma concentrations of above inflammatory cytokines in both patients and controls and determined the cytokines in supernatant, which were performed using single ELISA. ELISA kits for the pro‐inflammatory cytokines were purchased from Neobioscience Inc, Shenzhen, China. Additionally, the inflammatory cytokines were determined in plasma from the mice models using multiplex assay (RayBiotech). The plasma concentrations of ANA and anti‐dsDNA antibodies were purchased from Alpha Diagnostic using single ELISA. All samples were measured in duplicate.

### 2.8 Real‐time polymerase chain reaction

Total RNA was drawn from PBMCs using TRIzol (Invitrogen). Qualified RNA from each individual patient and control was reversely transcribed to cDNA (Bio‐Rad). Primers for IL‐38 and the internal control β‐actin are summarized in Table S1. Relative quantification of the gene transcript for IL‐38 was determined with an SYBR Green PCR Kit from TaKaRa on an Applied Biosystems 7900 Real‐Time PCR System as reported previously.^12^

### 2.9 Statistical analysis

Parametric and non‐parametric statistical analyses were used for calculating the mean ± standard deviation (SD) and median (P_25_, P_75_) according to the type of data, respectively. Comparisons between the groups were made using the Student t test or Mann‐Whitney *U* test where appropriate. For multiple variables’ comparison, the ANOVA statistical test was selected. With regard to the correlation studies, Spearman's rank correlation test was used. *P* < .05 was significant.

## 3 RESULTS

### 3.1 IL‐38 in relation to clinical and laboratory characteristics in patients with SLE

**FIGURE 1:**
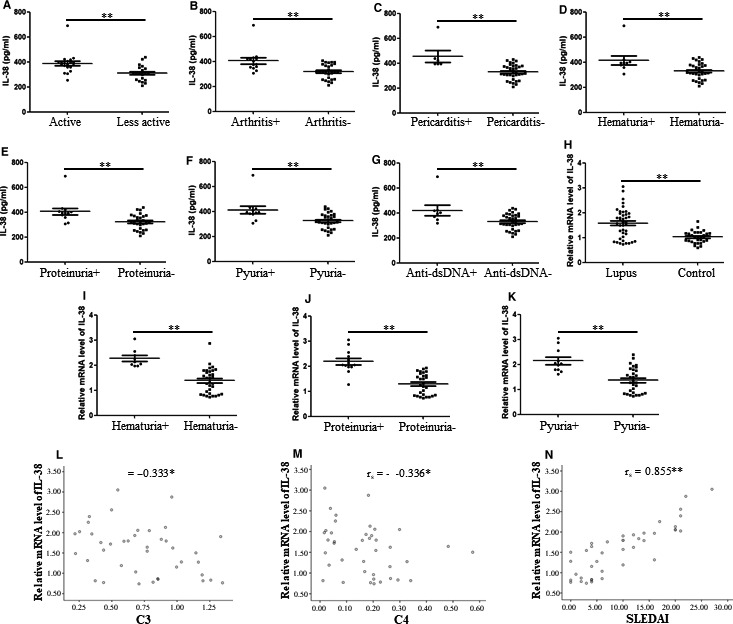
Correlation between IL‐38 and clinical and laboratory characteristics of patients with systemic lupus erythematosus (SLE). Levels of IL‐38 in plasma and peripheral blood mononuclear cells were discussed in patients with SLE (N = 41) and 31 healthy controls by enzyme‐linked immunosorbent assay and real‐time PCR. A, Patients with active disease revealed higher plasma levels of IL‐38 as compared with patients with less active disease. B‐G, Patients with features including arthritis, pericarditis, haematuria, proteinuria, pyuria and positive anti‐dsDNA showed higher plasma IL‐38 when opposed to patients without the features. H, Patients showed elevated mRNA expression of IL‐38 as compared with controls. I‐K, Patients with clinical features including haematuria, proteinuria, and pyuria revealed higher IL‐38 mRNA expression than patients without the features. L‐N, IL‐38 mRNA expression was related to C_3_, C_4_ and SLE Disease Activity Index. Mann‐Whitney *U* test was selected with respect to comparison between two groups, and correlation analysis was conducted by Spearman's non‐parametric test. *P < .05; **P < .005

In the previous study, we discussed elevated plasma IL‐38 in patients with SLE. To further elucidate the association of plasma IL‐38 and clinical and laboratory features in patients with SLE and the potential of plasma IL‐38 as a biomarker for SLE, we evaluated the relation of IL‐38 and the parameters and disease activity. Patients with SLE with active disease activity (N = 20) displayed significantly elevated plasma expression of IL‐38 as compared with patients with less active disease activity (N = 21) (386.92 [357.69‐406.30] vs 310.55 [256.56‐350.54] pg/mL, *P* < .005) (Figure 1A). Patients with positive clinical and laboratory features including arthritis, pericarditis, haematuria, proteinuria, pyuria and anti‐dsDNA showed strongly elevated plasma IL‐38 when compared with the patients without the features (Figure 1B‐G). Correlation analysis indicated that plasma IL‐38 significantly correlated with expression of C_3_ (*r*_s_ = −0.402, *P* < .05), C_4_ (*r*_s_ = −0.421, *P* < .05) and SLEDAI (*r*_s_ = 0.647, *P* < .005). Other parameters did not show a significant relationship of plasma IL‐38 with patients with SLE (Table 2; Table S2). Furthermore, to validate the dysregulated expression of IL‐38 in patients with SLE, we determined the IL‐38 mRNA expression in PBMCs of patients with SLE. We found that mRNA levels of IL‐38 were significantly higher in patients with SLE (N = 41) compared with those in healthy controls (N = 31, *P* < .005, Figure 1H). Subgroup analysis showed that patients with haematuria, proteinuria and pyuria features revealed elevated mRNA levels of IL‐38 as compared with the patients without the features (all *P* < .005, Figure 1I‐K). The levels of IL‐38 were negatively related to expression of C_3_ (*r*_s_ = −0.333, *P* < .05, Figure 1L) and C_4_ (*r*_s_ = −0.336, *P* < .05, Figure 1M). It is notable that mRNA levels of IL‐38 are strongly related to SLEDAI score (*r*_s_ = 0.855, *P* < .005, Figure 1N). Other parameters did not report a significant relationship of IL‐38 mRNA levels with patients with SLE (data not shown). These data suggest up‐regulated levels of IL‐38 in patients with SLE and are correlated with the disease activity.

**TABLE 2:** Correlation between plasma levels of IL‐38 and laboratory parameters in systemic lupus erythematosus patients

Parameters	Coefficient (*r*_s_)
C_3_	−0.402*
C_4_	−0.421*
IgA	−0.150
IgM	0.217
IgG	0.020
ESR	0.079
SLEDAI	0.647**

Abbreviations: ESR, erythrocyte sedimentation rate; SLEDAI, Systemic Lupus Erythematosus Disease Activity Index.

* *P* < .05;

** *P* < .005.

### 3.2 Plasma concentrations of inflammatory cytokines in patients with SLE and correlation with IL‐38

**FIGURE 2:**
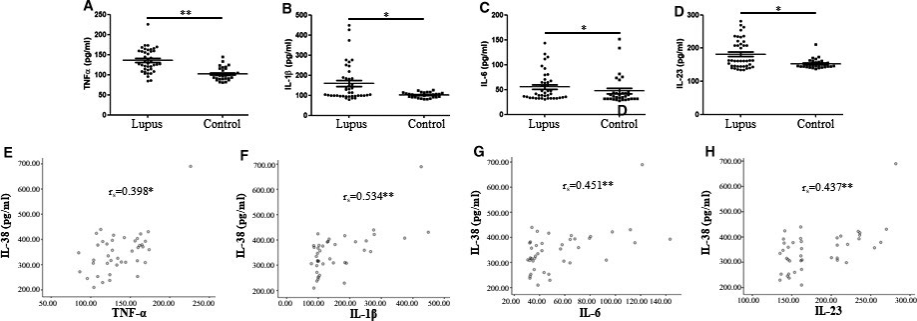
Inflammatory cytokines in systemic lupus erythematosus (SLE) patients and relation to IL‐38. Plasma levels of inflammatory cytokines TNF‐α, IL‐1β, IL‐6 and IL‐23 were tested by enzyme‐linked immunosorbent assay in 41 patients with SLE and 31 healthy controls. A‐D, All cytokines in plasma of patients with SLE were higher when compared with controls. Difference of cytokines between patients and controls was determined by Mann‐Whitney *U* test. E‐H, Plasma IL‐38 significantly correlated to levels of TNF‐α, IL‐1β, IL‐6 and IL‐23. Correlation analysis was conducted by Spearman's non‐parametric test. *P < .05; **P < .005

Inflammatory cytokines have been demonstrated to contribute to SLE pathogenesis.^3^ Plasma concentrations of TNF‐α, IL‐1β, IL‐6 and IL‐23 in patients with SLE were tested in our study. Findings revealed that the above inflammatory cytokines were up‐regulated in patients with SLE (N = 41) as compared with those in controls (N = 31), respectively (Figure 2A‐D). Because the study reported abnormal concentrations of IL‐38 and inflammatory cytokines, it is interesting to evaluate the association of IL‐38 and the cytokines. The results suggested that plasma levels of IL‐38 were significantly correlated with expression of TNF‐α (*r*_s_ = 0.398, *P* < .05), IL‐β (*r*_s_ = 0.534, *P* < .005), IL‐6 (*r*_s_ = 0.451, *P* < .005) and IL‐23 (*r*_s_ = 0.437, *P* < .005) (Figure 2E‐H).

### 3.3 Impact of IL‐38 on inflammatory cytokines in PBMCs from patients with SLE

**FIGURE 3:**
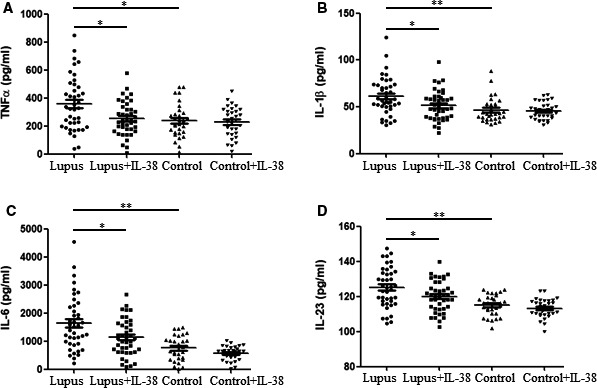
Impact of IL‐38 on inflammatory cytokines in peripheral blood mononuclear cells from patients with systemic lupus erythematosus (SLE). Peripheral blood mononuclear cells from patients with SLE (N = 41) and healthy volunteers (N = 31) were treated with recombinant human IL‐38 (200 ng/mL) for 24 h and then stimulated with LPS (1 μg/mL) for 6 h. Finally, supernatant was determined for TNF‐α, IL‐1β, IL‐6 and IL‐23 expression by enzyme‐linked immunosorbent assay (A‐D). Each symbol represents an individual patient or healthy volunteers. Non‐parametric test was used for analysis among different groups. *P < .05; **P < .005

Figure 2 shows the results had significant association with IL‐38 and inflammatory components. Therefore, it is possible that IL‐38 may regulate the cytokines and affect SLE pathogenesis. To test the hypothesis, PBMCs from patients with SLE (N = 41) and healthy controls (N = 31) were first stimulated with IL‐38 or without IL‐38 and then admitted LPS stimulation. The findings showed that TNF‐α expression in supernatant was strongly down‐regulated in patients with SLE after IL‐38 stimulation compared with that in those without IL‐38 stimulation (*P* < .05, Figure 3A). IL‐38 stimulation did not affect the expression of TNF‐α in the supernatant of PBMCs isolated from healthy controls (Figure 3A). Interestingly, we also determined a higher expression of TNF‐α in the supernatant from patients as compared with that from controls when PBMCs were not treated with IL‐38 (*P* < .05, Figure 3A), demonstrating the elevated expression of IL‐38 in patients with SLE. With respect to IL‐1β, IL‐6 and IL‐23 expression in the supernatant from patients with SLE under stimulation with IL‐38 or not, we found that these cytokines were strongly down‐regulated in patients with SLE after being stimulated by IL‐38 (Figure 3B‐D). The cytokines in supernatant from the patients with SLE strongly displayed a higher expression opposed to those from controls without IL‐38 stimulation. Cytokine expression in healthy controls was not significantly different before or after IL‐38 stimulation (Figure 3B‐D).

### 3.4 IL‐38 injection protects mice from serologic manifestations of lupus in C57BL/6 mice

**FIGURE 4:**
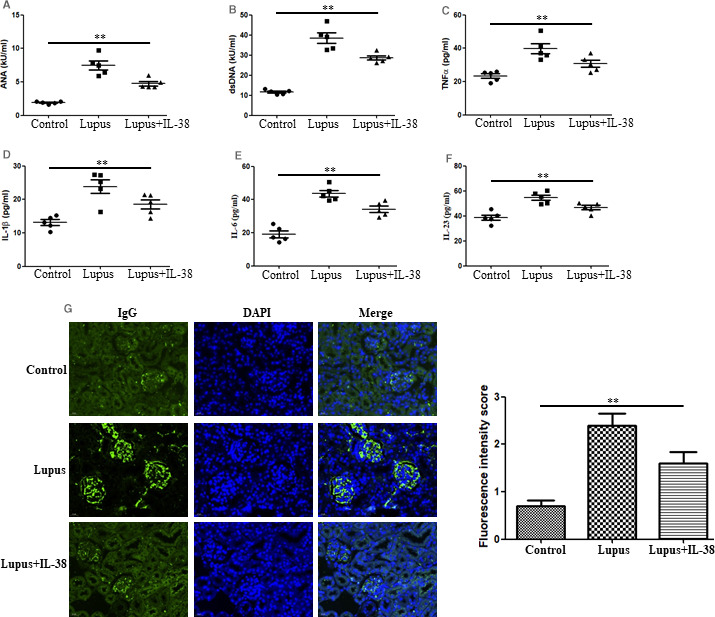
IL‐38 injection protects mice from serologic manifestations of lupus in C57BL/6 mice. Three groups of mice (n = 5/group, control mice, lupus mice and lupus + IL‐38 mice) were treated with pristane, saline or IL‐38 as described in Section 2. A‐B, Antinuclear antibody (ANA), anti‐dsDNA antibody, (C‐F) TNF‐α, IL‐1β, IL‐6 and IL‐23 expression in plasma were determined by enzyme‐linked immunosorbent assay at the 24th week. G, Deposition of IgG in kidney was evaluated by direct immunofluorescence method at the 24th week. Data were presented as mean ± SD. **P < .005. Differences among three groups of data were discussed by ANOVA

Compared with the control group, expression of ANA and anti‐dsDNA antibodies was significantly increased in pristane‐induced lupus mice (Figure 4A‐B). Of note, IL‐38 administration significantly reduced pristane‐induced ANA and anti‐dsDNA antibody levels. To evaluate the role of IL‐38 in circulating inflammatory biomarkers related to lupus and to confirm the abnormal expression of the inflammatory cytokines in patients with SLE we noted, the plasma levels of TNF‐α, IL‐1β, IL‐6 and IL‐23 were tested in pristane‐induced lupus mice treated with or without IL‐38. The results showed that plasma levels of TNF‐α (Figure 4C), IL‐1β (Figure 4D), IL‐6 (Figure 4E) and IL‐23 (Figure 4F) were significantly up‐regulated in pristane‐induced lupus mice compared with those in the control group. However, all the inflammatory cytokines were down‐regulated after IL‐38 treatment. Moreover, the glomerular IgG deposition was significantly reduced in IL‐38‐treated mice compared with that in the pristane‐induced lupus group (Figure 4G).

### 3.5 Injection of IL‐38 relieved nephritis and skin inflammation in C57BL/6 mice

**FIGURE 5:**
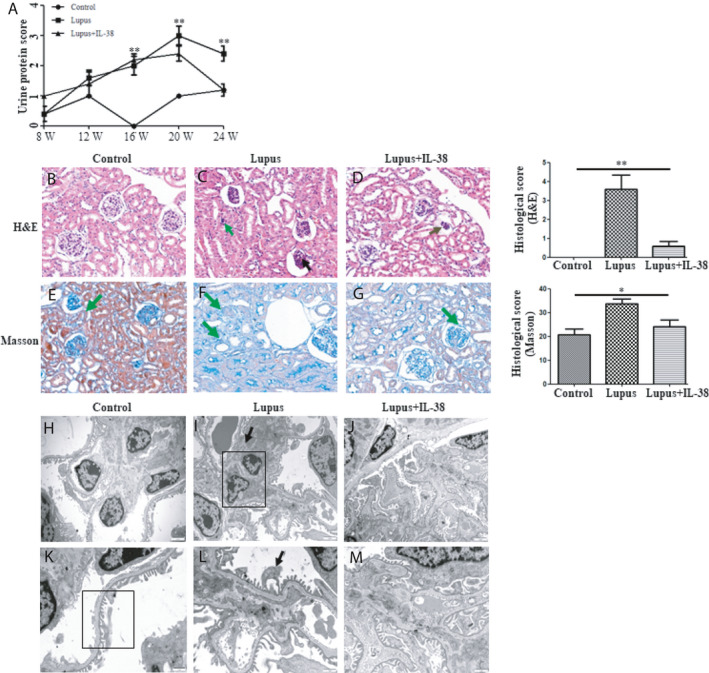
IL‐38 injection reduced the development of lupus renal disease in C57BL/6 mice. Three groups of mice (n = 5/group, control mice, lupus mice and lupus + IL‐38 mice) were treated with pristane, saline or IL‐38, and urine protein score and kidney morphology/ultrastructural evaluation of mice were measured. A, Urinary protein was tested at weeks 8, 12, 16, 20 and 24. B‐G, Photomicrographs showed glomerular, tubule, interstitium and medulla by haematoxylin and eosin (H&E) and Masson staining at the 24th week. Semi‐quantitative analysis of histological damage (H&E, ×400; Masson, ×400). H‐M, Transmission electron microscopy evaluated the kidney ultrastructure of three groups of mice at the 24th week. Bars H‐J, 2 um (×8000); K‐M, 1 um (×15 000). *P < .05; **P < .005 in the comparison among the three groups. Differences among three groups of data were discussed by ANOVA

Urinary protein was tested to examine for renal injury. There were low levels of urine protein score in the control group from the 8th week to the 24th week. On the contrary, pristane injection aggravated production of proteinuria, by which the urine protein score in both the lupus group and the lupus + IL‐38 group was significantly higher compared with that in the control group both at the 16th week and at the 20th week (*P* < .005, Figure 5A). The urine protein score in the lupus group and the lupus + IL‐38 group was comparable. Urine protein score in the lupus + IL‐38 group was significantly reduced after injection of IL‐38 compared to that in the lupus group (*P* < .005) at the 24th week and was similar to the score in control mice (Figure 5A). It is known that lupus mice develop inflammatory skin lesions such as hair loss in the dorsal neck region. The severity of skin lesions was evaluated before and after IL‐38 or saline injection. As shown in Figure S1, the mice in the lupus group treated with saline showed severe skin lesions at the 24th week, whereas mice in the lupus + IL‐38 group displayed reduced skin lesions after treatment with IL‐38. Mice in the controls showed no skin lesions before and after treatment with saline.

To discuss the effects of IL‐38 treatment on histopathology of the kidney, tissues of the kidney were collected at the 24th week. Histological examination was revealed by H&E (Figure 5B‐D) and Masson staining (Figure 5E‐G). As the control group shows, there were normal features of glomeruli and proximal and distal tubules. The capsule of the kidney tissues was intact, demarcation of the cortex and medulla was clear, and there was no capillary basement membrane thickening with inflammatory exudation and no findings of inflammatory cell infiltration and fibrous tissue proliferation in the renal interstitium. By contrast, the lupus group showed abnormal glomerular structure in the cortex; glomerular atrophy; innate immune cell necrosis with nuclear pyknosis, fragmentation and enlarged renal capsule volume; dilatation of renal tubule; and thinning of renal tubular wall. Moreover, there was epithelial degeneration, uneven cytoplasmic staining and exfoliation, dissolution of nucleus in epithelial cells from renal tubule, lymphocyte infiltration and fibrous connective tissue proliferation in the renal interstitium as well as protein casts in the collecting tubule from the medulla. In the lupus + IL‐38 group, IL‐38 injection can correct severe kidney injuries.

By ultrastructural analysis, the kidneys of the control mice showed clear structure, by which podocyte processes combined with capillary endothelium and basement membrane to form a filtration barrier. There were abundant mitochondria in the cytoplasm of renal tubular epithelial cells, arranged in a long columnar shape at the base, and the structure of the intramembranous fold was clear (Figure 5H,K). Conversely, damage was apparent in the kidneys of lupus mice, demonstrated by podocyte fusion, mesangial cell proliferation, deposits in the mesangial matrix and existence of vacuoles and autophagosomes in the cytoplasm of renal tubular epithelial cells (Figure 5I,L). Remarkably, the ultrastructural evaluation of lupus mice treated with IL‐38 reduced the effects of pristane‐induced lupus (Figure 5J,M).

## 4 DISCUSSION

In our previous study, we found higher expression of IL‐38 in patients with rheumatoid arthritis (RA), which also correlated to RA disease activity, showing the potential to be a disease marker for RA and might involve in disease development.^12^ Similarly, we observed elevated plasma expression of IL‐38 in patients with SLE; however, its clinical association and disease manifestation need elucidation. Furthermore, to evaluate the possibility of plasma IL‐38 as a disease marker for SLE still requires discussion. In the current study, we analyse IL‐38 expression in 41 patients with SLE and discuss in detail its relationship with patients’ clinical and laboratory features. Plasma IL‐38 is significantly related to features including arthritis, pericarditis, haematuria, proteinuria, pyuria and expression of C_3_ and C_4_. On the contrary, plasma levels of IL‐38 did not relate to rash; alopecia; fever; reduced platelet; antibodies ANA, anti‐Sm, anti‐SSA, anti‐SSB and anti‐rRNA; expression of IgA, IgM and IgG; or ESR. The experiments indicated that expression of IL‐38 correlated with some laboratory values and clinical manifestations of SLE. When the patients were divided into active disease group (SLEDAI > 10) and less active disease groups (SLEDAI < 10), the active group displayed elevated plasma levels of IL‐38. It is notable that IL‐38 expression significantly correlated with SLEDAI, suggesting that plasma IL‐38 correlated with disease activity and might be a potential disease marker for SLE. Although the plasma concentration was observed up‐regulated in patients with SLE, we tested its expression in PBMCs to prove observed results within plasma. Our data revealed elevated mRNA levels of IL‐38 in PBMCs in patients with SLE when opposed to controls, confirming elevated expression for IL‐38 in patients with SLE. Collectively, our experiments indicated that IL‐38 levels were elevated within SLE and may affect the development of this disorder.

Several lines of evidence reported that inflammatory cytokines destroyed the immunologic balance in lupus, including TNF‐α, IL‐1β, IL‐6 and IL‐23.^17^, ^18^, ^19^ Patients with SLE reported elevated serum concentration for TNF‐α,^16^ and TNF‐α signalling contributed to lupus development in lupus‐prone mice.^20^ TNF‐α genetic polymorphisms were strongly related to renal disorders, haematological manifestation and elevated prevalence of positive anti‐dsDNA antibody in patients with SLE.^21^ Additionally, serum expression for IL‐1β was strongly increased in patients with SLE,^22^ and IL‐6 regulated lupus nephritis development.^23^ IL‐23 receptor deficiency in lupus mice showed attenuated nephritis, accompanied by reduced accumulation of inflammatory cells in the kidney.^24^ We observed significantly higher plasma concentration for the above four inflammatory cytokines in patients with SLE in our study, demonstrating that these pro‐inflammatory components are dysregulated in SLE and may perform significantly in the pathogenesis of SLE.

In our study, we reported elevated expression of inflammatory cytokines in patients with SLE. Interestingly, our data also indicated that up‐regulated IL‐38 significantly correlated with disease activity. Therefore, what is the clear role of IL‐38 in lupus? Or what is the function of IL‐38 as an anti‐inflammatory inhibitor?^25^ To answer the question, we first evaluated the association of plasma IL‐38 and inflammatory components and found that IL‐38 concentration positively correlated with the cytokines, suggesting that IL‐38 might affect lupus aetiology through regulating pro‐inflammatory cytokines. To test the hypothesis, recombinant human IL‐38 was then adopted to stimulate PBMCs of patients with SLE, showing that IL‐38 can effectively down‐regulate the synthesis of these inflammatory components. To date, available evidence suggests that IL‐38 overexpression suppressed synthesis of IL‐6 and IL‐1β in PBMCs^26^ and inhibited synthesis of IL‐6, TNF‐α and IL‐23 in the THP‐1 monocytic cell line after IL‐38 overexpression.^27^ Therefore, it is warranted that is there a feedback loop for the inflammatory cytokines and IL‐38 in autoimmune disorders? Future designs are necessary to assess effects of pro‐inflammatory cytokines on IL‐38 expression for this hypothesis. It is known that IL‐38 and IL‐37 belong to the IL‐1 family cytokine with anti‐inflammatory activities.^28^ IL‐37 affects TNF‐α, IL‐1β and IL‐6 generation.^29^ In turn, these cytokines are able to promote the synthesis of IL‐37.^29^, ^30^ Thus, it is reasonable to elucidate that IL‐38 levels were increased in patients with SLE, related to activity of disease, because there is a positive feedback loop for elevated expression of IL‐38 by inflammatory cytokines like IL‐37. Additionally, reduced generation of the pro‐inflammatory components within the present study implied that IL‐38 may be important for suppression of inflammatory response in SLE, and elevated expression by immune reactions might come from the results of inflammatory disease self‐limiting. We found that IL‐38 did not affect expression of the four cytokines regarding cytokine generation in PBMCs from controls, indicating that the anti‐inflammatory activity of IL‐38 might only be applicable for the inflammatory phase.

Several studies have indicated the roles of IL‐38 in inhibition of autoimmune disease development (eg arthritis, psoriasis and lupus).^27^, ^31^ IL‐38 gene knockout mice treated with K/BxN serum revealed significant arthritic features such as increased disease activity up‐regulated synthesis of IL‐1β and IL‐6 expression in the joints.^31^ Collagen‐induced arthritic mice injected with IL‐38 down‐regulated clinical inflammatory scores and reduced the synthesis of IL‐23 and TNF‐α.^27^ Moreover, psoriatic patients reported an elevated expression of IL‐38 in skin biopsies after secukinumab treatment.^9^ On the contrary, release of IL‐38 was strongly reduced by IL‐22, IFN‐γ and IL‐36γ stimulation in human keratinocytes. The effects of IL‐38 on psoriasis development were demonstrated through IL‐38 injection into imiquimod‐induced psoriatic mice, showed down‐regulated levels of loricrin, CXCL8, CXCL20 and IL‐6, and ameliorated the manifestations, such as reduction of acanthosis and dermal inflammatory infiltrate.^9^ Furthermore, injection of IL‐38 into lupus‐prone mice strongly ameliorated clinical symptoms, for instance, reduced proteinuria, leukocyturia and skin lesions. Levels of IL‐22 in mice serum were down‐regulated after IL‐38 injection.^32^ IL‐38 injection inhibited phosphorylation of ERK1/2, P38 MAPK and the subunit P65 of NF‐κB in human dermal microvascular endothelial cell (HDMEC).^9^ It is known that MAPK (ERK1/2, p38) and NF‐κB signalling pathways are of importance in lupus pathogenesis.^33^, ^34^ These cascade proteins are able to regulate synthesis of inflammatory components. For example, classical NF‐κB signalling knockout in lupus‐prone mice strongly down‐regulated levels of IL‐1α, IFN‐γ and IL‐6 in the kidneys.^33^ Normalizing ERK1/2 in lupus‐prone mice reduced expression of IL‐1β, TNF‐α and IL‐6 and relieved development of lupus.^35^, ^36^ Therefore, it is possible that inhibiting the activity of downstream signallings by IL‐38 may cause less inflammatory component generation in PBMCs during inflammatory response, which will further result in inhibition of dysregulated immunity, and prevent development of lupus.

To confirm the role of IL‐38 in generation of inflammatory cytokines, we conducted the lupus mice model by injection of C57BL/6 mice with pristane. The findings showed that wild‐type mice that were injected with pristane at the 24th week had severe features of lupus, such as high score of urine protein, damaged histological examination of kidney sections, skin lesions and elevated levels of ANA, anti‐dsDNA and IgG deposition. On the contrary, IL‐38 administration to pristane‐induced lupus mice reduced the effects by pristane. It is notable that IL‐38 injection significantly reduced the levels of inflammatory cytokines IL‐1β, TNF‐α, IL‐6 and IL‐23 in plasma, demonstrating that IL‐38 is able to protect mice from lupus development, and has a negative role in regulating inflammatory cytokines both in vitro and in vivo. Considering the correlation between histopathology of the kidney, SLE and IL‐38, the current study found a reduced IgG renal deposition in lupus mice treated with IL‐38, and the plasma ANA and anti‐dsDNA antibody were reduced as well after IL‐38 administration, indicating that IL‐38 may suppress the production of autoantibodies in autoimmunity for mice. In another study by Chu et al, MRL/lpr mice showed a decreased percentage of splenic CD3 + CD4‐CD8‐ double‐negative (DN) T cells after IL‐38 treatment.^32^ It is known that DN T cells are a subpopulation of T cells that are highly expressed in patients with SLE and may be involved in tissue damage.^37^ DN T cells are able to promote IgG and anti‐DNA antibody generation in patients with SLE.^38^, ^39^ Therefore, a reduced percentage of DN T cells by IL‐38 treatment may help to down‐regulate the autoantibody accumulation in lupus.

In conclusion, this study indicates that IL‐38 expression correlated with activity of SLE and took part in the development of lupus by regulating inflammatory cytokine generation.

## CONFLICT OF INTEREST

None.

## AUTHOR CONTRIBUTION

**Wang‐Dong Xu:** Writing‐review & editing (equal). **Lin‐chong Su:** Writing‐review & editing (equal). **Xiao‐Yan Liu:** Investigation (supporting). **Jia‐Min Wang:** Software (supporting). **Zhi‐Chao Yuan:** Methodology (supporting). **Zhen Qin:** Investigation (supporting). **Xi‐Ping Zhou:** Formal analysis (supporting). **An‐Fang Huang:** Writing‐review & editing (equal).

## Supporting information

Fig S1

Table S1

Table S2

## Data Availability

Datasets are available from the corresponding author on reasonable request.
