# Supplementary figures and images for: IL‐38: A novel cytokine in systemic lupus erythematosus pathogenesis
Source: J Cell Mol Med. 2020 Oct 20;24(21):12379–89. doi: 10.1111/jcmm.15737 (PMC7686966; doi:10.1111/jcmm.15737)

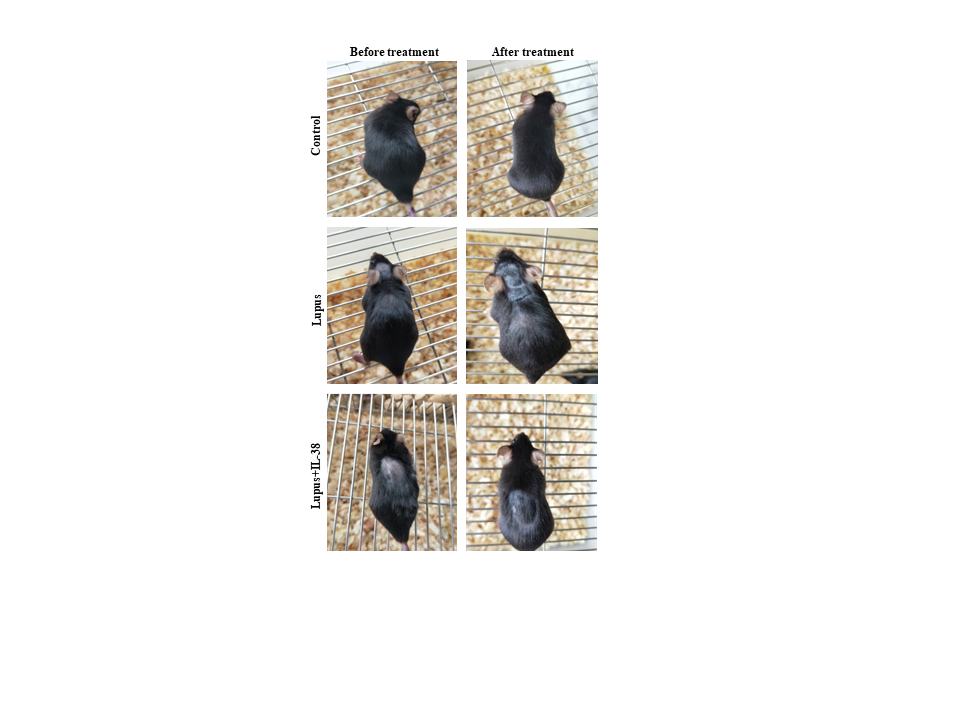

Supplement: Supplementary file 1 — Fig S1 [file JCMM-24-12379-s001.tif]
