# Supplementary material for: IL‐38: A novel cytokine in systemic lupus erythematosus pathogenesis
Source: J Cell Mol Med. 2020 Oct 20;24(21):12379–89. doi: 10.1111/jcmm.15737 (PMC7686966; doi:10.1111/jcmm.15737)
Supplement: Supplementary file 2 — Table S1 [file JCMM-24-12379-s002.docx]

Supplementary table 1 Primer pairs for real-time PCR.

| Gene | Forward (5’-3’) | Reverse (5’-3’) |
| --- | --- | --- |
| IL-38 | CCTCCCCATGGCAAGATACT | GCCAAGCCTCTGTTAGGAAG |
| β-actin | CTCCCTGGAGAAGAGCTACGAGC | CCAGGAAGGAAGGCTGGAAGAG |
