# Supplementary material for: IL‐38: A novel cytokine in systemic lupus erythematosus pathogenesis
Source: J Cell Mol Med. 2020 Oct 20;24(21):12379–89. doi: 10.1111/jcmm.15737 (PMC7686966; doi:10.1111/jcmm.15737)
Supplement: Supplementary file 3 — Table S2 [file JCMM-24-12379-s003.docx]

Supplementary table 2 Comparison of plasma IL-38 in different clinical and laboratory characteristics in systemic lupus erythematosus patients.

| Characteristics | Status | IL-38 (pg/ml) | P value |
| --- | --- | --- | --- |
| Disease duration | + | 325.15 (265.90-392.89) | >0.05 |
|  | - | 365.04 (316.86-395.54) |  |
| Rash | + | 367.59 (312.45-411.12) | >0.05 |
|  | - | 347.12 (285.05-393.39) |  |
| Alopecia | + | 373.29 (340.48-406.30) | >0.05 |
|  | - | 318.13 (265.90-392.32) |  |
| Fever | + | 340.64 (235.41-395.51) | >0.05 |
|  | - | 353.97 (306.14-393.96) |  |
| Reduced platelet | + | 379.85(236.91-415.56) | >0.05 |
|  | - | 347.12 (307.19-392.23) |  |
| ANA | + | 365.03 (315.71-395.83) | >0.05 |
|  | - | 310.55 (301.84-384.33) |  |
| Anti-Sm | + | 352.13 (311.18-375.95) | >0.05 |
|  | - | 353.97 (301.83-395.20) |  |
| Anti-SSA | + | 357.15 (309.43-378.68) | >0.05 |
|  | - | 341.02 (303.57-394.58) |  |
| Anti-SSB | + | 309.43 (252.95-523.51) | >0.05 |
|  | - | 356.65 (306.66-393.67) |  |
| Anti-rRNP | + | 374.21 (314.69-398.23) | >0.05 |
|  | - | 325.15 (298.37-392.82) |  |
